# High CO_2_ decreases the long‐term resilience of the free‐living coralline algae *Phymatolithon lusitanicum*

**DOI:** 10.1002/ece3.4020

**Published:** 2018-04-16

**Authors:** Laura Sordo, Rui Santos, Isabel Barrote, João Silva

**Affiliations:** ^1^ Marine Plant Ecology Research Group Centre of Marine Sciences (CCMAR) University of Algarve Faro Portugal

**Keywords:** calcification, coralline algae, long‐term, ocean acidification, photosynthesis, photosynthetic pigments, *Phymatolithon lusitanicum*, respiration, southern Portugal

## Abstract

Mäerl/rhodolith beds are protected habitats that may be affected by ocean acidification (OA), but it is still unclear how the availability of CO_2_ will affect the metabolism of these organisms. Some of the inconsistencies found among OA experimental studies may be related to experimental exposure time and synergetic effects with other stressors. Here, we investigated the long‐term (up to 20 months) effects of OA on the production and calcification of the most common mäerl species of southern Portugal, *Phymatolithon lusitanicum*. Both the photosynthetic and calcification rates increased with CO_2_ after the first 11 months of the experiment, whereas respiration slightly decreased with CO_2_. After 20 months, the pattern was reversed. Acidified algae showed lower photosynthetic and calcification rates, as well as lower accumulated growth than control algae, suggesting that a metabolic threshold was exceeded. Our results indicate that long‐term exposure to high CO_2_ will decrease the resilience of *Phymatolithon lusitanicum*. Our results also show that shallow communities of these rhodoliths may be particularly at risk, while deeper rhodolith beds may become ocean acidification refuges for this biological community.

## 1 INTRODUCTION

The increase of atmospheric CO_2_ projected by IPCC (2014) and consequent ocean acidification (OA) will cause major shifts on seawater chemistry. The lowering of the saturation state and carbonate concentration on seawater is expected to affect the ability of marine calcifiers to form their carbonate skeletons or shells (Kroeker, Kordas, Crim, & Singh, 2010). OA is also likely to affect photosynthesis due to the changes of the relative proportions of CO_2_ and ${\text{HCO}}_{3}^{-}$_,_ the two possible substrates for photosynthesis (Hurd, Hepburn, Currie, Raven, & Hunter, 2009; Martin, Cohu, Vignot, Zimmerman, & Gattuso, 2013). However, the variability in results among studies has prevented the scientific community to deliver a clear message on how OA will affect calcifiers in a near future (Gattuso, Mach, & Morgan, 2012).

The contribution of an ecosystem to the global carbon cycle is driven by the balance between carbon production and consumption and between calcium carbonate production and dissolution (Gattuso, Pichon, & Frankignoulle, 1995). Changes in the water chemistry as a consequence of pH decrease are expected to disrupt the ocean's carbon sequestration capacity and affect photosynthesis and calcification, both of which use DIC as substrate (Martin & Hall‐Spencer, 2017). However, information on the potential of marine ecosystems to mitigate the effect of increasing anthropogenic CO_2_ and the carbon storage capacity of photosynthetic calcifiers such as coralline algae is yet scarce (van der Heijden & Kamenos, 2015). Coralline algae are of particular interest to investigate as they conduct both photosynthesis and calcification simultaneously (Martin et al., 2013).

Beds of unattached red coralline algae (mäerl/rhodoliths) are important carbon sinks and nursery grounds for commercial species, being also major players in the global carbon cycle through the production of CaCO_3_ sediment (van der Heijden & Kamenos, 2015; McCoy & Kamenos, 2015). These habitats are found from the poles to the tropics, and can form extensive beds of 20,900 km^2^ in the tropical south‐west Atlantic (Amado‐Filho et al., 2012). In spite of their global distribution and importance, rhodolith beds have received less attention with respect to other more geographically restricted ecosystems such as coral reefs. Mäerl beds are globally distributed habitats especially sensitive to OA and global warming because of their slow growth rates and the high solubility of their high‐Mg calcite skeletons (McCoy & Kamenos, 2015; Williamson, Najorka, Perkins, Yallop, & Brodie, 2014). In a future ocean, filamentous algae are expected to be better competitors than coralline algae (Short, Kendrick, Falter, & McCulloch, 2014). The degradation and replacement of mäerl beds by fleshy algae communities is likely to affect the world's carbon cycle, with wide repercussions, including the fishery industry (Brodie et al., 2014).

Laboratory experiments have revealed mixed and sometimes contradictory responses of the photosynthesis, calcification, and respiration of coralline algae to OA, even among closely related species. Positive, negative, and parabolic responses have been observed (Martin et al., 2013), hampering the withdrawal of reliable conclusions and thus complicating the prediction of future consequences of OA on coralline algae. The variability in results have been attributed to length of experimental exposure (see Ragazzola et al., 2012, 2013), differences in algal physiology, methodologies used (gas bubbling vs. acid‐base additions, e.g.,), and/or pre‐experimental period (see Hurd et al., 2009).

Several authors have highlighted the importance of long‐term experiments in evaluating the responses of coralline algae to OA (e.g., Martin et al., 2013; McCoy & Kamenos, 2015; Ragazzola et al., 2013). Long‐term studies can reveal very different results with respect to short‐term ones (McCoy et al., 2016) and provide important information on the potential for physiological acclimation (see Hurd et al., 2009; Martin et al., 2013; Ragazzola et al., 2013). Nevertheless, the response of coralline algae to OA has mainly been investigated through short‐term experiments (Martin et al., 2013). The longest OA experimental studies conducted with coralline algae lasted for a year (Martin & Gattuso, 2009; Martin et al., 2013), using the species *Lythophyllum cabiochae* (crustose morphology). In contrast, the longest experiments with mäerl species ranged from 1 to 3 months (e.g., Burdett et al., 2012; Kamenos et al., 2013; Noisette, Duong, Six, Davoult, & Martin, 2013; Noisette, Egilsdottir, Davoult, & Martin, 2013; Ragazzola et al., 2012) to a maximum of 10 months (see Ragazzola et al., 2013).

Ragazzola et al. (2013) compared the responses of the species *Lithothamnion glaciale* (mäerl morphology) after 3 and 10 months of high‐CO_2_ treatment and obtained different results with time. After 3 months, algae cultivated under high CO_2_ maintained their growth rate but reduced their cell wall thickness, while, after 10 months, the cell wall thickness was maintained, but there was a reduction in the growth rates. The authors concluded that long‐term experiments provide a better analog for understanding the organism's response to OA and a more accurate projection of future impact. Long‐term experiments, where the synergistic effects of light, temperature and other factors are also investigated, are essential to understand how ecosystems will respond to global change.

Few studies have investigated the complex and tightly linked processes of photosynthesis, calcification, and respiration on coralline algae (e.g., Martin et al., 2013; Noisette, Duong, et al., 2013; Noisette, Egilsdottir, et al., 2013). These three metabolic processes are able to alter the pH of seawater, thus modifying the carbon speciation (Hurd et al., 2009). The increase in pH during photosynthesis increases the CaCO_3_ saturation state and consequently fosters calcification, while respiration decreases pH, hindering calcification (see Gao et al., 1993; Martin et al., 2013). A better understanding of how these processes are inter‐related under an OA scenario is required to elucidate how mäerl species will respond to global environmental changes (Hurd et al., 2009; Martin et al., 2013).

The current challenge in OA research is to understand how whole ecosystems react to a range of climate‐related stressors (Dupont & Pörtner, 2013a). Recent model projections have demonstrated that regionally distinct patterns of complex oceanic change are evident, and considerations of multistressor patterns can help to guide laboratory and field studies (Boyd, Lennartz, Glover, & Doney, 2015). However, most experimental studies do not consider the interactions encompassing environmental factors such as irradiance, temperature, or nutrients, which are determinant on the algae's respiration, photosynthesis, and calcification. Thus, more realistic manipulations, in which natural fluctuations in these factors are included, will reveal more true‐to‐life responses (Boyd et al., 2016).

Long‐term field experiments incorporating environmental variability (e.g. McCoy, 2013), as well as organismal adaptation, may provide unparalleled insights into the effects of global change (Kroeker, Kordas, & Harley, 2017) on coastal ecosystems. Moreover, the better understanding of the environmental effects in combination with the sublethal effects reported in laboratory experiments will help to forecast the long‐term ecological effects of global change (Kamenos, Perna, Gambi, Micheli, & Kroeker, 2016). Still, experimental studies are not usually complemented by the monitoring of the seasonality of natural communities, leading to an important gap between experimental and community‐scale studies (McCoy & Kamenos, 2015).

The synergistic effects of environmental stressors such as temperature (see Vásquez‐Elizondo & Enríquez, 2016) and high irradiance (Gao & Zheng, 2010; Yildiz, Hofmann, Bischof, & Dere, 2013) are expected to aggravate and/or accelerate the detrimental effect of OA, and are determinant factors for mäerl/rhodolith beds, especially for those located at more shallow areas and intertidal zones.

Mäerl beds in Algarve (southern Portugal) are mainly composed of unattached, monospecific branches of the nongeniculate coralline red algae *Phymatolithon lusitanicum* (Peña et al., 2015). Recent molecular analyses have confirmed *P. lusitanicum* as a new species (Peña et al., 2015). The species *Lithothamnion corallioides* and *Phymatolithon calcareum* are also present on the Algarve mäerl beds, but their occurrence is rare (Carro, López, Peña, Bárbara, & Barreiro, 2014; Peña et al., 2015). These two species are especially abundant in the British Isles and Brittany, but they have been gradually replaced by *Phymatolithon lusitanicum* in Galicia (NW Spain) (Carro et al., 2014). *P. lusitanicum* is particularly abundant in subtidal mäerl beds of the Atlantic Iberian Peninsula, in Galicia (4–13 m) and Algarve (15–25 m), but it has also been detected in Ireland (intertidal zone to 6 m), in the Alborán Sea (40–48 m) and in the Balearic Islands (54–64 m) (Peña et al., 2015). The largest rhodoliths of *P. lusitanicum* in the Iberian Peninsula have been found in the southernmost locations and show their thickest morphology in Algarve (Carro et al., 2014; *P. lusitanicum* referred as *Phymatolithon sp3* in Peña et al., 2015).

The effect of OA on the mäerl species from southern Portugal is so far unknown. The objective of this study was to investigate, for the first time, the long‐term (20 months) effects of OA on the photosynthesis and calcification of *Phymatolithon lusitanicum* (Peña et al., 2015), the main species of the most southwestern mäerl/rhodolith beds in Europe. The effect of OA on respiration was also assessed.

## 2 MATERIAL AND METHODS

### 2.1 Biological material

The thalli were collected by SCUBA diving in Armação de Pêra, Faro (N 37°011′.650″/W‐8°19′.034″). This rhodolith bed covers an extension of about 3 km^2^, from 13 to 25 m depth. The algae were sampled at 22 m depth, immediately transferred to a cool box maintained at in situ temperature and transported to the CCMAR field station at the Ria Formosa natural park. The thalli were gently cleaned to remove the excess of epiphytes and placed in aquaria with filtered seawater, where they were kept under controlled conditions during all the experiment.

### 2.2 Experimental system

The experimental system used in this study is described in detail in Sordo, Santos, Reis, Shulika, and Silva (2016). Briefly, seawater is pumped from an adjacent coastal lagoon in front of CCMAR field station and passes through a preliminary mechanical filtration and through two in‐line filters of 10–20 and 5 μm before entering a preliminary 2,000 L reservoir. Here, seawater is continuously bubbled (compressed air at ambient conditions) with air stones to reach equilibrium with the atmosphere and further routed through two 16 and 8W UV filters before being distributed into three 200 L head tanks.

The CO_2_‐air mix bubbling system is located in an isolated container where photoperiod and temperature are adjusted seasonally. This system works in an open circuit with low water flow rates. CO_2_ injection is controlled by a solenoid valve coupled to an Infrared Gas Analyzer (WMA‐4 IRGA; PPSystems, USA), a PID controller (PID330; TEMPATRON, UK) and an air‐flushing equilibrator (Frankignoulle, Borges, & Biondo, 2001). A mix of food‐grade CO_2_ and ambient air is injected into the three 200 L head tanks using an air pump. This is a novel OA experimental design, conceived to run long‐term experiments (Sordo et al., 2016).

### 2.3 Experimental design

Based on the CO_2_ emission perspectives of the International Panel on Climate Change (IPCC IS92) (IPCC, 2000), two enrichment levels of *p*CO_2_ = 550 μatm (2050 projected scenario) and *p*CO_2_ = 750 μatm (2100 projected scenario) were simultaneously tested together with a control level without enrichment (*p*CO_2_ ~ 400 μatm). A total of 270 thalli (90 per *p*CO_2_ level) were labeled with numbered plastic tags attached with nylon fishing wire and randomly distributed by the eighteen 25‐L aquaria and maintained at the different CO_2_ levels for 20 months. The aquaria were regularly cleaned to control the growth of turf algae. The growth of the thalli was followed during the experiment using the nondestructive buoyant weight technique. In addition, unlabeled thalli were kept in the aquaria for metabolic measurements.

Photoperiod was adjusted using a timer to the desired L:D (light and dark, hr) according to natural fluctuations. It varied from 10:14 in December to 15:9 in June. The ambient light source consisted of two 12W led strings, green and white, above the aquariums. The photosynthetic photon flux density was kept at ca. 8 μmol photons m^−2^ s^−1^. These values were calculated based on field‐collected data from an annual sampling cycle. Air and water temperatures were controlled with an AC apparatus and water chillers (Sunsun HYH‐0.5 D‐C, China).

### 2.4 Seawater parameters

The seawater and air temperature were monitored every 5 min using HOBO temperature loggers (Onset Corp.). Salinity (CO310 conductivity meter, VWR, USA), pH (Orion 8103SC pH meter, Thermo scientific, USA), temperature (Roth digital thermometer, Hanna, EU), and dissolved oxygen (Symphony SB90M5, VWR*, USA,* accuracy ± 0.2 mg/L; ±2%) were measured in each aquarium on a biweekly basis. Total alkalinity (TA) was measured at different points of the system: the source 2,000 L tank, the three head 200 L tanks, and the 25 L aquariums where the algae were kept. The *p*CO_2_ at each treatment was recorded every 20 min by a gas analyzer (WMA‐4; PPSystems, USA).

In parallel to this study, we continuously monitored the temperature and PAR levels in the natural sampling site using autologging sensors. During the experiment, temperature was gradually adjusted to the values observed under natural conditions in the field.

### 2.5 Photosynthesis, respiration, and calcification

Net photosynthesis, respiration, and calcification rates of algae exposed to the different *p*CO_2_ treatments were determined three times during the experiment (at 0, 11, and 20 months of treatment) through short time incubations. After 11 months (March 2013), incubations were conducted at different irradiance levels (0, 8, 45, 195, and 450 μmol photons m^−2^ s^−1^), and the effect of high CO_2_ on the respiration and photosynthetic rates of algae was determined. Light intensities were selected based on the average levels of PAR determined in the natural beds and on parameters from previously determined light‐response curves for this species (Sordo et al., 2016). At 0 and 20 months (April 2012 and December of 2013, respectively), light incubations were performed at the saturating irradiance of 200 μmol photons m^−2^ s^−1^.

Preweighted algae individuals were placed in 500 ml Erlenmeyer flasks (*n* = 4) filled to the top and sealed to prevent gas loss by leakage. For the photosynthetic and calcification measurements, 20 g of mäerl was incubated for 1 hr per light intensity and CO_2_ level. Dark respiration (*R*_d_) and calcification (*G*_d_) were measured using 40 g of mäerl incubated for two and a half hours per CO_2_ level. To guarantee robust respiration results, the algae were left in the dark for 30 min prior to the incubations.

Samples for dissolved oxygen and total alkalinity (TA), to calculate the calcification rates of algae (Steller, Hernandez‐Ayon, Riosmena‐Rodriguez, & Cabello‐Pasini, 2007), were collected at the beginning and at the end of the incubations. Temperature, pH, and salinity were measured. Dissolved oxygen was analyzed by direct spectrophotometry as described in Labasque, Chaumery, Aminot, and Kergoat (2004). Briefly, a calibration curve was performed with six different concentrations of KIO_3_ (0, 40, 80, 160, 240 and 320 μmol/L). Cl_2_Mn 3 mol/L, NaOH 8 mol/L, and NaI 4 mol/L were added to the calibration curve as well as to the water samples right after collection. After acidification with H_2_SO_4_ 10 mol/L, the iodometric reaction was measured by direct spectrophotometry at 466 nm (Beckman Coulter DU‐650).

The net community photosynthesis or net photosynthesis (NP) and dark respiration (*R*_d_) were calculated from the difference between initial and final concentrations of oxygen, normalized by the incubation time, the volume of the chamber, and the fresh weight of the incubated thalli according to the formula;

$$\text{NP}\;\text{or}\;R_{d}\;\left(\mu {\text{molO}}_{2}\;{\text{gFW}}^{-1}\;{\text{hr}}^{-1}\right)\;=\;\left(\left[O_{2}\right]\;\text{fin}-\left[O_{2}\right]\;\text{in}\right)\;\times \;V/\left(\text{FW}\;\times \;T\right)$$

where [O_2_] is the oxygen concentration (μmol/L), *V* is the volume of the chamber (L), FW is the fresh weight of the incubated thalli (g), and *T* is the incubation time (hours).

The alkalinity (TA) anomaly technique (see Smith & Key, 1975) was used to determine the calcification rates (Steller et al., 2007). When 1 mole of CaCO_3_ precipitates, TA decreases in the ratio of 1:2 (see Wolf‐Gladrow, Zeebe, Klaas, Koertzinger, & Dickson, 2007). Total alkalinity was determined using the Gran titration method as in Lewis and Wallace (1998). Seawater was sampled at the beginning and end of the incubations and at different points of the system in 100 ml Winkler bottles and immediately poisoned with 20 μl of saturated mercuric chloride. TA was measured in subsamples of 80 ml which were titrated using an open cell automatic titration system comprising an Orion 8103SC pH electrode calibrated on the National Bureau of Standards (NBS) scale, and a computer‐driven basic titrator (Metrohm 794 dosimat titrator, Switzlerland, EU) using an acid titrant of HCl 0.5 mol/L. The values were corrected using Certified Reference Materials (CRMs, Batch No. 121 and 126) supplied by A. Dickson (Scripps Institution of Oceanography, USA). The carbonate chemistry of seawater samples was determined from measured pH, TA, temperature, and salinity using the software CO_2_SYS (Lewis & Wallace, 1998) with the constants of Mehrbach, Culberson, Hawley, and Pytkowicz (1973) (refitted by Dickson & Millero, 1987), that is, dissolved inorganic carbon (DIC) and saturation state of aragonite (the saturation state of high Mg‐calcite is closer to aragonite than calcite). Light and dark calcification rates (*G*) were calculated from the increment in total alkalinity, normalized by the incubation time, the volume of the chamber, and the fresh weight of the incubated thalli according to the formula described in Steller et al. (2007):

$$G\;\left(\mu \text{mol}\;{\text{CaCO}}_{3}\;{\text{gFW}}^{-1}\;{\text{hr}}^{-1}\right)\;=\;-\left(\Delta \text{TA}\right)\;\times \;V/\left(2\;\times \;\Delta t\;\times \;\text{FW}\right)$$

where ΔTA is the difference between initial and final total alkalinity (μmol CaCO_3_/L), *V* is the incubation volume (L), Δ*t* is the incubation time (hours), and FW is the fresh weight of the sample (g).

### 2.6 Calcium carbonate and organic matter content (HCl acidification)

After 20 months of high CO_2_ exposure, organic matter and CaCO_3_ composition in the algae were determined by eliminating CaCO_3_ through acidification (see Steller et al., 2007). Twenty‐four individual rhodoliths per CO_2_ level (*n* = 24) were weighed and then dried to constant weight. After this step, each rhodolith was placed in 25 ml of 5% HCl which was changed every 24 hr until no bubbles were observed and all CaCO_3_ was dissolved. Samples were rinsed with distilled water and dried to constant weight in a drying oven at 60°C. Organic matter and CaCO_3_ were determined by subtraction from the preacidified dry weight.

### 2.7 Calcification/growth (buoyant weight technique)

The calcification/growth of algae was determined using the buoyant weight (BW) technique, firstly proposed for coral growth by Jokiel, Maragos, and Franzisket (1978) and also used in coralline algae (e.g., Short et al., 2014 and Steller et al., 2007). A total of 270 thalli (90 per CO_2_ level) were tagged at the beginning of the experiment. The individual weight of each thallus was determined after 7, 12, 16, and 20 months, by suspending the sample in a beaker filled with filtered seawater by a nylon string attached to an electronic balance (Sartorius 0.1 mg).

The weight of CaCO_3_ at each sampling time (Wcc) was calculated as (Steller et al., 2007);

$$\text{Wcc}\;=\;\text{Wb}\;\times \;{\left(1-\text{Dw}\;\times \;{\text{Dcc}}^{-1}\right)}^{-1}$$

where Wcc is the dry weight of the CaCO_3_, Wb is the buoyant weight of the algae, Dw is the density of the seawater (1.03 g/cm^3^), and Dcc is the density of CaCO_3_ (2.71 g/cm^3^). Replacing the densities of seawater and CaCO_3_, the following equation is obtained;

Wcc=1.61Wb

The total growth was calculated by dividing the difference between the initial and the final buoyant weight by the initial weight of each replicate. The calcification rate was calculated by dividing the total growth by the number of days between measurements.

### 2.8 Determination of photosynthetic pigments

Samples for pigments were collected at the end of the experiment. Algae were frozen in liquid nitrogen and stored at −80°C until analysis. For chlorophylls and carotenoids, approximately 1.5 g FW was ground in liquid nitrogen, immediately extracted in 5 ml of 100% acetone and centrifuged for 5 min at 2,000 ×g and 4°C. Chlorophylls *a* and *d* concentrations were calculated according to Ritchie (2008);

$$\text{Chl}\;a\;\left(g/m^{3}\right)\;=\;-0.3319\;A_{630}-1.7485\;A_{647}\;+\;11.9442\;A_{664}-1.4306\;A_{691}$$

$$\text{Chl}\;d\;\left(g/m^{3}\right)\;=\;-0.5881\;A_{630}\;+\;0.0902\;A_{647}-0.1564\;A_{664}-11.0473\;A_{691}$$

where:

A_630_—absorbance at 630 nm, A_647_—absorbance at 647 nm, A_664_—absorbance at 664 nm, A_691_—absorbance at 691 nm. The limits of detection (and inherent error, ±95% confidence limit) for the Chl equations were <0.03 g/m^3^ (Ritchie, 2008).

Carotenoids concentration was calculated according to Torres et al. (2014);

$$\text{Carotenoids}\;\left(\mu g/\text{ml}\right)\;=\;\left(1,000\;\times \;A_{470}-1.90\;\times \;\text{Chl}\;a\right)/214$$

where:

$$A_{470}-\text{absorbance}\;\text{at}\;470\;\text{nm}$$

Phycoerythrin (PE) and phycocyanin (PC) content were determined from 1 g of fresh thalli ground in liquid nitrogen and extracted in 2 ml of 0.1 mol/L phosphate buffer (pH 6.5) at 4°C after centrifugation (Heraeus Megafuge 16R, Thermo scientific, USA) at 4696 ×g for 40 min. PE and PC concentrations were determined according to Sampath‐Wiley and Neefus (2007);

$$\text{Phycocyanin}\;\left(\text{PC}\right)\;\left(\text{mg}/\text{ml}\right)\;=\;0.154\;\left(A_{618}-A_{730}\right)$$

$$\text{Phycoerythrin}\;\left(\text{PE}\right)\;\left(\text{mg}/\text{ml}\right)\;=\;0.1247\;[\left(A_{564}-A_{730}\right)-0.4583\;\left(A_{618}-A_{730}\right)]$$

where:

A_618_—absorbance at 618 nm, A_730_—absorbance at 730 nm, A_564_—absorbance at 564 nm.

All absorbances were measured in a Beckman Coulter DU‐650 spectrophotometer.

### 2.9 Statistical analyses

SigmaPlot (version 11.0) software package was used to perform all statistical analysis. The effects of CO_2_ on net O_2_ evolution under dark and ambient conditions were assessed with a Kruskal–Wallis analysis of variance (ANOVA on ranks) as data presented equal variance but did not meet the assumptions of normal distribution (Kolmogorov–Smirnov test) after a log and square root transformation. When significant differences were found, a post hoc test (Student–Newman–Keuls, SNK) was applied to explore differences between treatments. The effect of CO_2_ and light and CO_2_ and time of exposure on photosynthesis was assessed with separate two‐way ANOVA tests where normal distribution (Kolmogorov–Smirnov test) and equal variance (Levene's test) were verified. When *p* was significant (*p *<* *.05), ANOVA was followed by a post hoc test for multiple comparisons (Tukey's HSD).

The effects of CO_2_ on the calcification of the algae under dark and ambient conditions were assessed with a two‐way ANOVA when normal distribution (Kolmogorov–Smirnov test) and equal variance (Levene's test) were verified after a log transformation of the data. When *p* was significant (*p *<* *.05), ANOVA was followed by a post hoc test for multiple comparisons (Tukey's HSD). The effect of CO_2_ on the calcification with light, the effect of CO_2_ on the calcification with time, and the calcium carbonate content with CO_2_ were assessed with separate Kruskal–Wallis ANOVA on ranks as data presented an equal variance but did not meet the assumptions of normal distribution (Kolmogorov–Smirnov test) after a log and square root transformation. When significant differences were found, a post hoc test (Student–Newman–Keuls, SNK) was applied to explore differences between treatments.

The Pearson correlation coefficient was used to assess the linear dependence between photosynthesis and calcification after 11 months_,_ and between growth rate and total growth with CO_2_ and time.

The effect of CO_2_ on the pigment concentrations was analyzed using a one‐way ANOVA. When significant differences were found, a post hoc test (Student–Newman–Keuls, SNK) was applied to explore differences between treatments. As carotenoid data presented equal variance but did not meet the assumptions of normal distribution (Shapiro–Wilk test), data went through a square root transformation prior to analysis.

## 3 RESULTS

**Figure 1:**
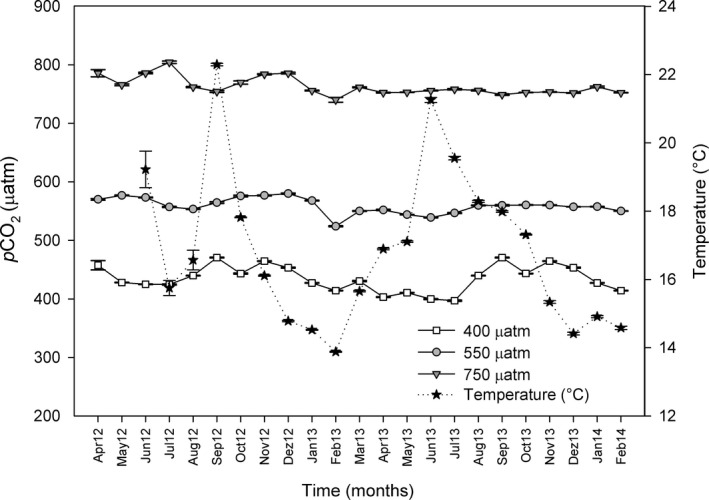
Monthly *p*CO_2_ average values (μatm ± standard error; *n* = 2204) from April 2012 (Apr12) to February 2014 (Feb14), under 400, 550, and 750 μatm pCO2 conditions, and monthly temperature values (°C  ±  standard error; *n* = 2,975) (from June 2012 to February 2014). The values were measured with an IRGA (WMA‐4, PP Systems, USA) every 20 min and an HOBO temperature logger (Onset Corp.) every 15 min

Salinity, temperature, and dissolved oxygen did not differ among CO_2_ treatments. During the 20 months of the experiment, salinity ranged from 34 to 38 psu and temperature fluctuated in a similar way as the natural beds (14–20°C) (Figure 1). Dissolved oxygen at the mesocosms remained constant (~6.7 mgO_2_/L). Total alkalinity (TA) was identical at different points of the experimental system both in CO_2_ and control treatments and ranged between 2,449 and 2,536 μmol/kgSW (Table 1).

**Table 1:** Carbonate chemistry for each CO_2_ concentrations (400, 550 and 750 μatm) after 11 and 20 months of high CO_2_. Total alkalinity (TA), salinity (Sal.), temperature (*T*) and pH were measured while dissolved inorganic carbon (DIC) and aragonite saturation state (Ω_arag_) were calculated using CO_2_SYS software. Values expressed as means ± standard error (*n* = 11)

Time	*p*CO_2_ (μatm)	TA (μmol/kgSW)	Sal. (psu)	*T* (°C)	pH	DIC (μmol/kgSW)	Ω_arag_
11 months	400	2461.76 ± 4.07	34.87 ± 0.02	15.64 ± 0.19	8.18 ± 0.01	2183.93 ± 7.48	3.09 ± 0.05
	550	2449.17 ± 2.67	34.84 ± 0.02	15.92 ± 0.12	8.10 ± 0.00	2215.27 ± 2.84	2.65 ± 0.03
	750	2468.31 ± 2.84	34.63 ± 0.16	15.74 ± 0.14	7.90 ± 0.03	2326.21 ± 11.02	1.81 ± 0.11
20 months	400	2513.72 ± 3.52	36.30 ± 0.00	17.00 ± 0.00	8.06 ± 0.00	2292.98 ± 3.48	2.53 ± 0.01
	550	2517.91 ± 3.99	36.40 ± 0.00	16.80 ± 0.00	8.01 ± 0.01	2323.36 ± 4.19	2.28 ± 0.01
	750	2536.66 ± 9.11	36.40 ± 0.00	17.05 ± 0.01	7.87 ± 0.01	2401.50 ± 9.10	1.76 ± 0.02

The mean CO_2_ values under control conditions ranged from 397 to 470 μatm of CO_2_, while the mean values for the intermediate CO_2_ treatment ranged from 539 to 577 μatm of CO_2,_ and the mean values for the high CO_2_ treatment ranged from 740 to 804 μatm of CO_2_ (Figure 1 and Table 1).

### 3.1 Effects of CO_2_ and time of exposure on photosynthesis and of CO_2_ on respiration. Photosynthesis and calcification relationship

**Figure 2:**
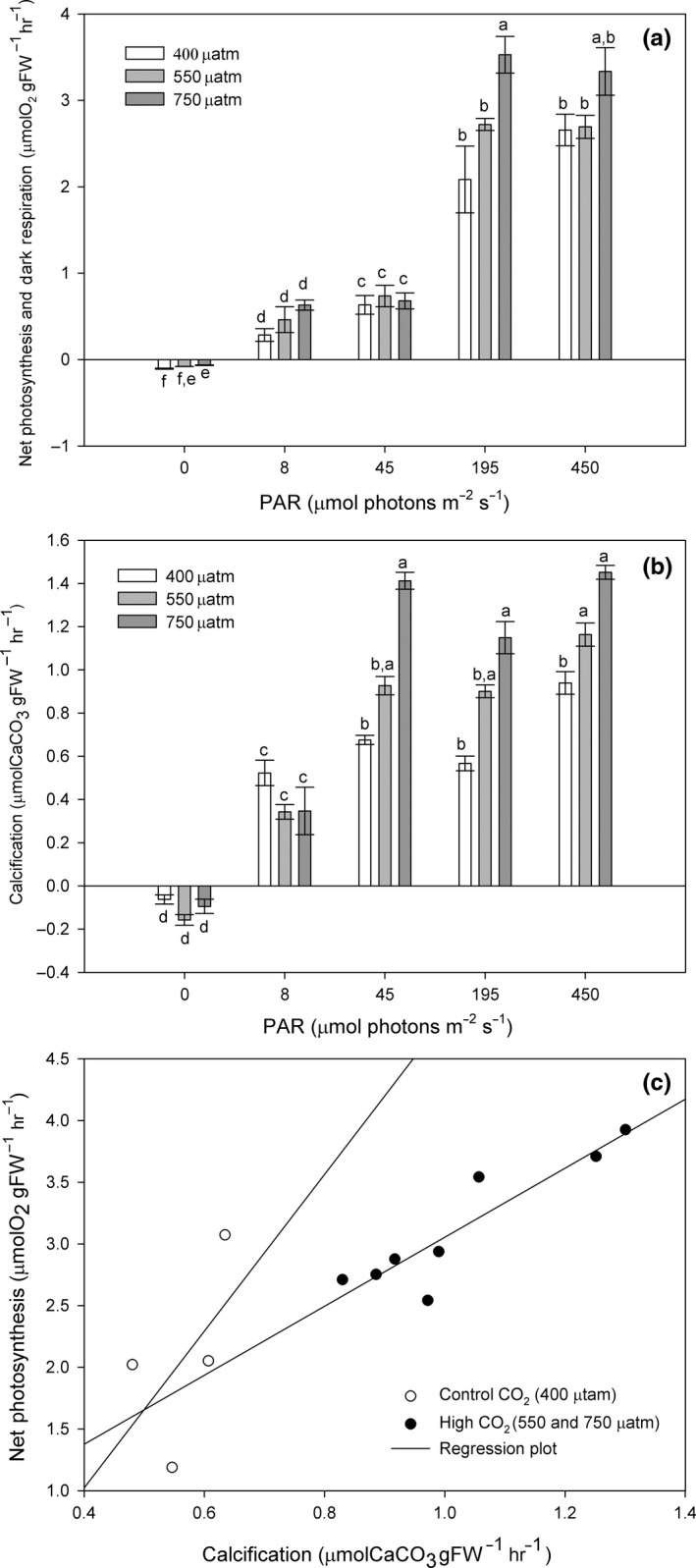
*Phymatolithon lusitanicum*. Photosynthesis, respiration, and calcification after 11 months of exposure at three different CO_2_ levels (400, 550 and 750 μatm) (a) Dark respiration and net photosynthesis as a function of light (8, 45, 195, and 450 μmol photons m^−2^ s^−1^), (b) net calcification as a function of light (0, 8, 45, 195, and 450 μmol photons m^−2^ s^−1^) results calculated using the total alkalinity anomaly technique, and (c) correlation of net photosynthesis and light calcification at 200 μmol photons m^−2^ s^−1^ under control and high CO_2_ conditions. Different letters indicate significant differences with CO_2_ and irradiance. Values are expressed as mean ± standard error (*n* = 4)

Photosynthetic rates increased significantly with increasing light (Figure 2a). As well, photosynthetic rates increased significantly with CO_2_ (*p* = <.001), but only at high levels of both light (195 and 450 μmol photons m^−2^ s^−1^) and CO_2_ (750 μatm). CO_2_ had also a significant positive effect (*p* = <.001) on dark respiration (Figure 2a). Control algae presented the highest respiration rates (0.1 μmolO_2_ gFW^−1^ hr^−1^) with respect to 550 μatm (0.08 μmolO_2_ gFW^−1^ hr^−1^) and 750 μatm of CO_2_ (0.07 μmolO_2_ gFW^−1^ hr^−1^).

After 11 months, net photosynthesis and calcification (Figures 2a, b) tend to increase together and were positively correlated (*r* = .69; *p* = .000000000963). However, this correlation was more accentuated under control conditions and decreased under high CO_2_ (Figure 2c).

**Figure 3:**
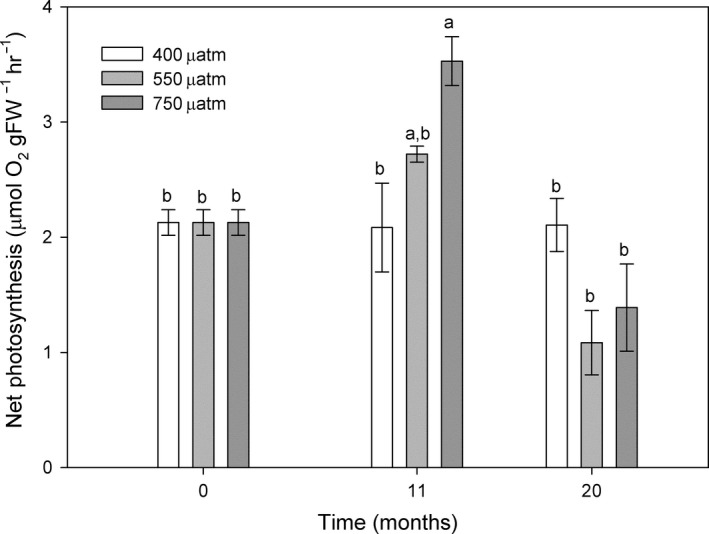
Net photosynthesis (μmolO_2_ gFW ^−1^ hr^−1^) of *Phymatolithon lusitanicum* at ~200 μmol photons m^−2^ s^−1^ (PAR) incubated for 0, 11, and 20 months under three different CO_2_ levels (400, 550, and 750 μatm). Different letters indicate significant differences with CO_2_ and time. Values expressed as mean ± standard error (*n* = 4)

The net photosynthesis of acidified algae at ~ 200 μmol photons m^−2^ s^−1^ changed significantly with time of exposure to high CO_2_ (*p *=* *.004) (Figure 3). After 11 months of treatment, the net photosynthetic rates were higher at both elevated CO_2_ levels and presented a significant increase under high CO_2_ conditions (750 μatm). However, after 20 months, this pattern was reversed, with respect to the results observed after 11 months. Algae from the high CO_2_ treatment showed a significantly decrease in photosynthetic rates with no differences from the initial results, and algae under intermediate CO_2_ conditions (550 μatm) decreased their rates. In contrast, algae kept at control conditions presented unaltered rates through time (*p *=* *.998) (Figure 3).

### 3.2 Effects of CO_2_ and time of exposure on calcification

After 11 months of high CO_2_ exposure, the calcification rates of *Phymatolithon lusitanicum* increased with light intensity. Algae increased their calcification rates with CO_2_ at irradiances of and above 45 μmol photons m^−2^ s^−1.^ Calcification in the dark was always negative (net dissolution) (Figure 2b), and there were no significant effects of CO_2_.

In contrast, the calcification rates, calculated using the alkalinity anomaly technique after 20 months at 200 μmol photons m^−2^ s^−1,^ were not significantly affected by CO_2_ concentration (*p* = .380). However, when comparing these results with the ones obtained after 11 months (see Figure 2b at 195 μmol photons m^−2^ s^−1^), only control algae kept constant and even increased their instant calcification rates at the end of the experiment (0.73 ± 0.10 μmolCaCO_3_ gFW^−1 ^hr^−1^), while acidified algae decreased their instant calcification rates after 20 months (0.86 ± 0.09 μmolCaCO_3_ gFW^−1 ^hr^−1^ under 550 μatm and 0.94 ± 0.11 μmolCaCO_3_.gFW^−1 ^hr^−1^ under 750 μatm).

**Figure 4:**
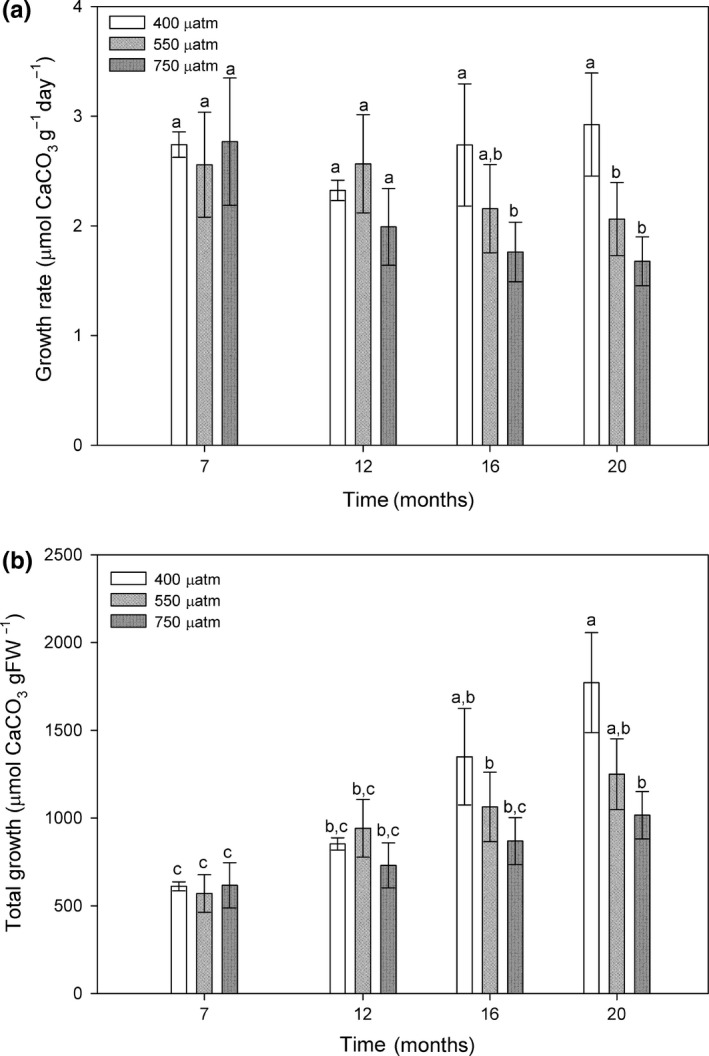
(a) Calcification/growth rates (μmolCaCO_3_ gFW ^−1^ day^−1^) and (b) total cumulative growth (μmolCaCO3/gFW) of *Phymatolithon lusitanicum* thalli cultivated under low light ambient irradiance (8 μmol photos m^−2 ^s^−1^) at three different CO_2_ levels (400, 550 and 750 μatm) after 7, 12, 16, and 20 months of high CO_2_. Results calculated using the buoyant weight technique. Different letters indicate significant differences with CO_2_ and time. Values expressed as mean ± standard error (*n* = 90)

The calcification rates of *Phymatolithon lusitanicum* maintained at low light levels of 8 μmol photos m^−2^ s^−1^ decreased significantly under exposure to high CO_2_ levels of 550 and 750 μatm in relation to control algae, but only after 16 months (Figure 4a). After nearly 2 years of treatment, control algae presented the highest accumulated growth (1771.62 μmolCaCO_3_/gFW), while under CO_2_ levels of 550 and 750 μatm algae presented a lower total growth (1249.73 and 1016.34 μmolCaCO_3_/gFW, respectively) with respect to control algae (Figure 4b).

Significant growth was observed in all CO_2_ treatments. Total growth rates were positively correlated with time under control (*r* = .265; *p* = .0000114), 550 μatm (*r* = .168; *p* = .00311) and 750 μatm conditions (*r* = .149; 0.0155). Only algae kept under control CO_2_ levels presented a positive correlation between growth rate and time (*r* = .0322; *p* = .6), while the growth rate of algae under 550 μatm (*r* = −.0504; *p* = .379) and 750 μatm (−0.116; *p* = .0598) presented a negative correlation with time. However, after 20 months of experiment, both the growth rate and the total growth of the algae decreased with CO_2_ and a significant negative correlation (*r* = −.202; *p* = .0155) was found. These results suggest that both CO_2_ and time of exposure have a negative effect on the growth/calcification of these algae.

The calcium carbonate content (gCaCO_3_/gDW) of *Phymatolithon lusitanicum* was not affected by CO_2_ concentrations (Kruskal–Wallis one‐way; *p *=* *.101), with mean values (±SE) of 3.25 ± 0.27 for control (400 μatm), 2.92 ± 0.20 for the 550 μatm treatment and 2.74 ± 0.21 for the 750 μatm CO_2_ treatment.

### 3.3 Effect of CO_2_ on the photosynthetic pigment concentration

Even if some bleaching was observed on the algae exposed to high CO_2_ (personal observations, not quantified), the photosynthetic pigments were not affected by CO_2_ concentration (Table 2). *Phymatolithon lusitanicum* presented higher concentrations of phycoerythrin than phycocyanin. Chlorophyll *d*, a pigment present in some red algae adapted to low light conditions, was not detected.

**Table 2:** Chlorophyll *a*, carotenoids, phycocyanin, and phycoerythrin pigment concentrations (mg/gFW) of *Phymatolithon lusitanicum* under 400, 550 and 750 μatm conditions after 20 months of high CO_2_ under low light ambient irradiance (8 μmol photons m^−2 ^s^−1^). Values expressed as mean ± standard error (*n* = 5). *p* Values show the significance of the effects of CO_2_ levels on pigment concentration

*p*CO_2_ (μatm)	Chlorophyll *a* (mg/gFW)	Carotenoids (mg/gFW)	Phycocyanin (mg/gFW)	Phycoerythrin (mg/gFW)
400	0.040 ± 0.006	0.016 ± 0.004	0.004 ± 0.000	0.122 ± 0.019
550	0.048 ± 0.004	0.014 ± 0.002	0.004 ± 0.000	0.153 ± 0.015
750	0.039 ± 0.002	0.016 ± 0.001	0.003 ± 0.000	0.097 ± 0.016
*p* Values	.901	.887	.446	.105

## 4 DISCUSSION

Our study shows the importance of establishing long‐term experiments to detect thresholds of resistance to OA, and reveals that time of exposure must be considered to predict how calcified macroalgae will respond to elevated CO_2_. To our knowledge, this is the longest OA experiment with mäerl coralline algae (20 months). Previous OA short time experiments with coralline algae have shown a variety of contrasting responses such as that growth rates decreased with high CO_2_, after 20 hr (Gao et al., 1993) or 1 month (Büdenbender, Riebesell, & Form, 2011; Hofmann, Yildiz, Hanelt, & Bischof, 2012; Johnson & Carpenter, 2012), and that this decrease is intensified under high light irradiance events (Gao & Zheng, 2010; Yildiz et al., 2013) or exacerbated with warning (Büdenbender et al., 2011; Johnson & Carpenter, 2012; *see* Martin & Hall‐Spencer, 2017 *for review*). On the other hand, other short time experiments have found no effect on the gross production, calcification or respiration after 3 weeks at different CO_2_ concentrations (Egilsdottir, Noisette, Noël, Olafsson, & Martin, 2013), or an increase in the coralline algae's growth rate (Ragazzola et al., 2013). High CO_2_ also seems to decrease the Mg concentrations on the carbonate that may reduce elasticity, thus changing the structural properties of algae (Ragazzola et al., 2016), and this decrease is exacerbated with temperature (Nash, Martin, & Gattuso, 2016).

Longer experiments showed sustained growth rates or no effect after 1 year (Martin et al., 2013), increases after 80 days (Kamenos et al., 2013), increases after 4 months with the highest growth rates under static pH 8.05 and lowest under fluctuating pH 7.65 (Roleda et al., 2015) or decreases after 3 months (Noisette, Duong, et al., 2013; Ragazzola et al., 2013) or 1 year (Martin & Gattuso, 2009) of high CO_2_ exposure.

*Phymatolithon lusitanicum* showed positive responses to high CO_2_ during the first year, but after 2 years, control algae presented the highest rates and accumulated growth with respect to acidified algae. During the first 11 months, both photosynthetic and calcification rates increased with CO_2_. However, after 20 months of treatment, photosynthesis and calcification decreased at both concentrations of 550 and 750 μatm. Similar CO_2_/time of exposure limits have been found with other coralline algae species, for example, after 1 year (Martin & Gattuso, 2009; Martin et al., 2013) three months (Noisette, Duong, et al., 2013) or between 3 and 10 months (Ragazzola et al., 2013). Previous research suggests that in a future high CO_2_ world, coralline algae will be able to sustain high production rates for some time, but losses on their structural integrity are likely to happen (Burdett et al., 2012; Kamenos et al., 2013; Martin et al., 2013; Ragazzola et al., 2012). The calcification under lowering saturation state of calcium carbonate represents additional energetic costs that may not be sustained in the long term, in spite of increasing photosynthetic rates, resulting in a decrease in the general resistance of the organism, facilitating disease development and bleaching and reducing the algae ability to resist boring by predators (Ragazzola et al., 2012) or to compete with fleshy algae (Short et al., 2014).

After 11 months of OA, the observed increase in both calcification and photosynthetic rates may be considered a mechanism to compensate low pH and carbonate saturation state levels. Kamenos et al. (2013) demonstrated that the structure of coralline algal is more sensitive to rate rather than magnitude of acidification. At low pH, coralline algae survived by increasing their calcification rates, but the authors detected weakness in the calcite skeletons. Even if acidified algae calcified more to compensate OA, they were under a constant dissolution/calcification process (see Dupont & Pörtner, 2013b) and grew less in the long term (20 months). We found that after the first 11 months, calcification and photosynthesis grew together; however, this correlation was stronger under control conditions, and in the long term, the net production, growth rates, and total growth of algae decrease with CO_2_.

Previous research under natural conditions has shown that in the northeast Pacific, crustose coralline algae (CCA) competitive interaction and community structure have changed over 30 years due to ocean acidification (McCoy, 2013). By examining morphological change and comparing individuals of four CCA species that coexist in this area over time, McCoy and Ragazzola (2014) found that all of them maintain less skeletal material than in the past but the specific response depends on the algae's morphology. Because results change not only with CO_2_, but also with time of exposure, long‐term experiments are mandatory to detect and predict the real effect that OA will have on coralline algae. So, even if algae are able to temporarily compensate the low pH by increasing their rates, they also dissolve more, and in the long term, this counterpoint has a negative effect on their overall growth.

Contrary to our observation that *Phymatolithon lusitanicum* respiration decreased with higher CO_2_, most authors have found that coralline algae's respiration rates do not change with high CO_2_ (Martin et al., 2013; Noisette, Duong, et al., 2013; Noisette, Egilsdottir, et al., 2013). In a previous study with *Phymatolithon lusitanicum,* we also found that respiration was unaffected by CO_2_ but positively affected by temperature (Sordo et al., 2016). Even if we included the natural fluctuations in temperature, these fluctuations were small and the effect of temperature was not investigated during this study. It is, however, known that the effect of ocean acidification is exacerbated with warming (Martin & Hall‐Spencer, 2017), and according to Vásquez‐Elizondo and Enríquez (2016), coralline algae physiology can more adversely affected by temperature than by CO_2_. However, few studies have investigated the simultaneous effect of temperature and high CO_2_ on the photosynthesis, respiration, and calcification of coralline algae (e.g., Martin et al., 2013; Noisette, Duong, et al., 2013; Noisette, Egilsdottir, et al., 2013).

When we compare the pigment level with other red coralline algae, the amount of chlorophyll *a* on *P. lusitanicum* was similar to the values found for the mäerl species *Lithothamnion corallioides* at 15 μmol photons m^−2^ s^−1^ (0.037–0.072 mg/gFW) but presented higher carotene concentrations (0.0085–0.00153 mg/gFW) (Noisette, Duong, et al., 2013). Other intertidal species incubated at higher irradiances presented higher pigment concentrations such as, for example, *Corallina elongata* (experiment at 30 μmol photons m^−2^ s^−1^) which presented a Chl*a* concentration of 0.42 mg/gFW (Egilsdottir et al., 2013) or *Corallina officinalis* (incubated at 50 μmol photons m^−2 ^s^−1^) (Yildiz et al., 2013) which presented a higher Chl *a* (0.24 mg/gFW), phycoerythrin (0.24–0.33 mg/gFW), and phycocyanin concentration (0.21–0.27 mg/gFW) with respect to the subtidal *P. lusitanicum*.

Even though we observed some bleaching on the acidified algae by the end of the experiment (personal observations), their pigment concentrations were unaffected by CO_2_. Different authors have also found that chlorophyll *a* (Egilsdottir et al., 2013; Martin et al., 2013; Noisette, Duong, et al., 2013; Yildiz et al., 2013), phycobiliprotein (Yildiz et al., 2013), and carotenoid concentrations (Gao & Zheng, 2010; Noisette, Duong, et al., 2013) are unaffected by high CO_2_. But in combination with light, Gao and Zheng (2010) found that chlorophyll *a* and phycobiliproteins of *Coralline sessilis* decrease. Yildiz et al. (2013) also found that that the phycobiliproteins decreased with CO_2_ and light, while Martin et al. (2013) did not find any difference in Chl *a* concentration under different light regimes and CO_2_ concentrations. However, pigments are expected to change with irradiance and further studies on the effect of CO_2_ and irradiance on pigment concentration are necessary.

The irradiance used during OA experiments to incubate the algae and during the short time measurements is a determinant factor in the results. Punctual increasing irradiance events might intensify the effect of OA on *Phymatolithon lusitanicum*, and shallow populations may be more susceptible to OA. On the other hand, deeper populations may be less vulnerable as at low ambient light and in dark conditions, there were no effects of OA on the instant photosynthesis and calcification. This was not the case of another long‐term experiment of 1 year with the species *Lithophyllum cabiochae*, whose photosynthesis decreased under ambient light conditions and increased CO_2_ (Martin et al., 2013).

This study emphasizes the need for more long‐term OA studies with coralline algae, where other variables can be tested simultaneously. We showed that results changed with time of exposure to high CO_2_. In the first 11 months, acidified algae increased their photosynthetic and calcification rates to compensate the low saturation state of the seawater. But after a 20‐month exposure, acidified algae could not compensate the corrosive condition of seawater and decreased both their photosynthetic and calcification rates. At the end of the experiment, acidified algae presented the same production rates than during the initial measurements but lower calcification rates, while control algae kept constant rates during the whole experiment and presented the highest accumulated growth.

The future outcome of mäerl beds from southern Portugal to OA will depend on other factors involved that are not easy to predict. *Phymatolithon lusitanicum* may survive with minor production losses until intermediate CO_2_ concentrations are reached (550 μatm, IPCC projections for 2050), but in the next century (750 μatm, IPCC projection for 2100), the species resilience to OA may decline seriously, and shallow population may be particularly susceptible to increasing high CO_2_. Deeper areas where rhodolith beds are exposed to low light may become ocean acidification refuges for this biological community.

## CONFLICT OF INTEREST

None declared.

## AUTHOR CONTRIBUTIONS

Laura Sordo wrote the manuscript and contributed with the design of the work and the acquisition, analysis and interpretation of data. Rui Santos contributed with the design of the work, interpretation of data, and revision of the manuscript giving a final approval to the version submitted. Isabel Barrote contributed with the design of the work, interpretation of data, and revision of the manuscript giving a final approval to the version submitted. João Silva contributed with the design of the work, the interpretation of data, and the revision of the initial drafts of the manuscript giving a final approval to the version submitted. This research is a contribution to the FCT‐funded project PTDC/MAR/115789/2009 (jmsilva@ualg.pt). The first author (lsnieves@ualg.pt) was supported by the FCT doctoral grant SFRH/BD/76762/2011 under the supervision of João Silva, Isabel Barrote and Rui Santos. All of the undersigned authors participated actively in the study, and none has any potential conflict of interest. All of the authors have read and approved the manuscript in its present form and have agreed to its submission. Both Laura Sordo and João Silva are Corresponding authors of this manuscript. Laura Sordo is the contact for the Editorial process (including Editorial Manager and direct communications with the office), and she is responsible for communicating with the other authors (Rui Santos, Isabel Barrote and João Silva) about progress, submissions of revisions and final approval of proofs.
